# A PET-CT study of the uptake of labeled aptamers [^18^F]FB-Gol1 and [^18^F]FB-GR20 in rat 101.8 glioblastoma model

**DOI:** 10.3389/fonc.2025.1713181

**Published:** 2025-12-15

**Authors:** Andrey A. Postnov, Igor N. Pronin, Nina B. Vikhrova, Diana B. Kalaeva, Elena V. Pyzhik, Alexey A. Lipengolts, Alexander V. Revishchin, Fatima M. Dzarieva, Yahya A. Sliman, Andrey V. Golovin, Vladimir A. Korshun, Vladimir A. Brylev, Vsevolod A. Skribitsky, Yulia A. Finogenova, Kristina E. Shpakova, Elena Yu. Grigorieva, Anna I. Alekseeva, Anna V. Smirnova, Alexey M. Kopylov, Galina V. Pavlova, Dmitry Yu. Usachev

**Affiliations:** 1 Federal State Autonomous Institution "N.N. Burdenko National Medical Research Center of Neurosurgery" of the Ministry of Health of the Russian Federation, Moscow, Russia; 2 The Lebedev Physical Institute of the Russian Academy of Sciences, Moscow, Russia; 3 National Research Nuclear University MEPhI, Moscow, Russia; 4 “N.N. Blokhin National Medical Research Center of Oncology” of the Ministry of Health of the Russian Federation, Moscow, Russia; 5 Institute of Higher Nervous Activity and Neurophysiology, Russian Academy of Sciences, Moscow, Russia; 6 Lomonosov Moscow State University, Moscow, Russia; 7 I.M. Sechenov First Moscow State Medical University, Moscow, Russia; 8 Shemyakin-Ovchinnikov Institute of Bioorganic Chemistry, Russian Academy of Sciences, Moscow, Russia; 9 Avtsyn Research Institute of Human Morphology of Federal State Budgetary Scientific Institution “Petrovsky National Research Centre of Surgery”, Moscow, Russia; 10 Moscow Clinical Scientific Center named after Loginov, Moscow, Russia

**Keywords:** Gol1 aptamer, GR20 aptamer, glioblastoma, glioma diagnostics, EGFR, rat 101.8 glioblastoma model, animal PET-CT

## Abstract

**Background/Objectives:**

The ability to predict the values of immunohistochemical (IHC) biomarkers noninvasively for brain tumors is an important diagnostic task, accelerating the medical decision-making process and reducing the burden on the patient. In this work, the epidermal growth factor receptor (EGFR) is considered as a biomarker, the expression of which is associated with accelerated division and proliferation of cancer cells. The aim of this work is to study the binding of [^18^F]FB-Gol1 and [^18^F]FB-GR20 aptamers to the rat 101.8 glioblastoma model using PET-CT.

**Methods:**

The tissue model of rat 101.8 glioblastoma was transplanted to Wistar rats (n=14). Rats with developed tumors underwent successive PET-CT examinations with [^18^F]FB-Gol1, [^18^F]FB-GR20 and [^18^F]FDG (n=11 completed), followed each time by MRI study (T1, T2, T1 with contrast enhancement). Time activity curves for both aptamers were analyzed. After the animals were euthanized, glial tumor tissue was taken for IHC tests to confirm EGFR expression.

**Results:**

Both [^18^F]FB-Gol1 and [^18^F]FB-GR20 were captured by the tumor within the first minutes after i/v administration. During one hour the accumulation in the tumor fell down to a quarter of the initial level. Both radiotracers showed no apparent signal in healthy tissue. The standardized maximum uptake value in the tumor was SUVt=0.44 ± 0.22 and 0.43 ± 0.20 for [^18^F]FB-GR20 and [^18^F]FB-Gol1, respectively. The metabolic volume of [^18^F]FB-GR20 and [^18^F]FB-Gol1 was also similar, 0.069 ± 0.056 cm^3^ versus 0.064 ± 0.053 cm^3^. At the same time, the metabolic volume of the tumor, measured by PET, turned out to be less than the volume of contrast enhanced tumor tissue on MRI and partially did not coincide with it in space. The radioactive label 4-[^18^F]-fluorobenzylazide alone injected separately does not accumulate in the tumor. Immunohistochemical analysis showed the presence of EGFR expression in rat 101.8 glioblastoma samples taken from animals.

**Conclusions:**

This study demonstrates that both [^18^F]FB-Gol1and [^18^F]FB-GR20 radiopharmaceuticals bind to the rat 101.8 glioblastoma and may serve as promising candidates for the development of a diagnostic radiotracers for selective diagnosis of EGFR expression in glial tumors.

## 1 Introduction

Glioblastoma, as one of the most malignant types of brain tumors, remains an in-curable disease with a poor prognosis and life expectancy of about 1 year after diagnosis (1–3). Attempt at early diagnosis and treatment of this type of neoplasm have not yet been successful. During recent years, new data about the tumor biology have been obtained based on modern immunohistochemical and molecular methods of the tumor structure analysis, identifying several of the most common biomarkers responsible for the prognosis of the disease and the response to drug therapy.

Positron emission tomography combined with computed tomography (PET/CT) is an important step towards minimally invasive examination of the tumor biology prior to its treatment, as the level of activity of various metabolic processes may be directly or indirectly related to the prognosis of the disease. The possibility of noninvasive detection of tumor biomarkers using PET/CT is an important diagnostic task that accelerates the medical decision-making process.

The targeted radionuclide diagnostics of various types of tumors is the fastest growing field of nuclear medicine with a wide range of possibilities. However, clinically significant results have not yet been achieved in the field of neuro-oncology.

The prospect of developing new targeted radiopharmaceuticals for PET/CT diagnosis and subsequent treatment (theranostics) of brain tumors may be especially relevant in the case of glioblastomas that do not respond well to standard treatments, including radiation and chemotherapy (4).

Aptamers, DNA or RNA oligonucleotide molecules that bind specifically to certain target molecules, are considered to be the promising agents for targeted diagnostics and therapy of oncological diseases (5). Aptamers have high specificity in neurooncology, as they are able to overcome the blood-brain barrier (BBB) through receptor-mediated transcytosis (6). Aptamers have demonstrated accumulation in the brain in animal models (7–9).

One of the important advantages of aptamers (compared to antibodies) is their low immunogenicity. Aptamers can specifically bind to proteins overexpressed in glioma cells, such as platelet-derived growth factor receptor β (PDGFRβ) and Axl receptor tyro-sine kinase, which are involved in the tumor growth and progression. For example, the RNA aptamer Gint4.T, which targets PDGFRß, inhibits signal transduction, reducing proliferation and migration of glioma cells. This property can be used to deliver therapeutic molecules such as anti-microRNA-10b (7).

Some researchers believe that the GL21.T aptamer, which targets Axl by suppressing the migration and invasion of tumor cells, can be combined with Gint4.T to enhance the therapeutic effect (8). Aptamers can be conjugated to drug-carrying nanoparticles or nanocomplexes to improve drug targeting. For example, Gint4.T was used to modify nanocomplexes with PI3K-mTOR inhibitors, resulting in significantly increased drug uptake and toxicity in glioma cells compared to “free” drugs (9).

Aptamer-coated exosomes loaded with chemotherapeutic drugs such as temozolomide showed an improved survival in glioma models with reduced systemic toxicity. Aptamers conjugated with a radiopharmaceutical can serve as novel imaging agents for PET/CT by selectively binding to different targets expressed by glioma cells, helping to detect tumors and differentiate them from normal brain tissue (10, 11).

In this work, the epidermal growth factor receptor (EGFR) is considered as a significant biomarker of the aggressive development of the tumor process, the overexpression of which is associated with accelerated division and proliferation of glioma cells (12). The choice of the Gol1 aptamer for testing on laboratory animals is due to its advantages over the most studied aptamer U2 (13, 14). The GR20 aptamer, which we studied previously, was chosen for comparison. It is obtained by deleting the secondary structure elements of the U2 aptamer, which not only makes it shorter and more accessible, but also adds more affinity for EGFR in “*in vitro*” experiments (15).

We assumed that both aptamers [^18^F]FB-GR20 and [^18^F]FB-Gol1, labeled with the radioactive label 4-[^18^F]-benzylazide, should selectively accumulate on PET scan in glioblastoma tissue with EGFR overexpression.

For the work, we chose the model of rat glioblastoma 101.8, which is as close as possible in its histological images to human glioblastoma, as we demonstrated previously (16).

The aim of this investigation is to study the uptake of aptamers [^18^F]FB-Gol1 and [^18^F]FB-GR20, labeled with 4-[^18^F]-benzylazide in laboratory animals with implanted glioblastoma 101.8, using PET/CT, which allows direct visualization of these tracers accumulation in the tumor.

## 2 Materials and methods

### 2.1 Experimental animals

Sexually mature male Wistar rats (Pushchino breeding facility, Russia, P = 55-65, n=14, weight =250–350 grams) were used in the study. The rat tissue 101.8 glioblastoma was chosen as a brain tumor model. Model was obtained from “Collection of experimental tumors of the nervous system and neural tumor cell lines” of Avtsyn Research Institute of Human Morphology of Federal state budgetary scientific institution “Petrovsky National Research Centre of Surgery”, Moscow, Russia. The technology of implementation of this model was described in details earlier (16, 17).

The crushed tumor of 0.8–1 million cells was transplanted from one animal to another by intracranial implantation in the area of the right hemisphere of the brain (from the bregma line: 2.0 mm laterally, 2.0 mm caudally) to a depth of 4.0 mm from the outer surface of the skull. After 10–14 days, the rats developed clinical symptoms of glioma.

Then the animals underwent MR- imaging with contrast enhancement to determine the tumor size. In case of visible accumulation of the contrast agent in the volume of more than 2 mm^3^, each animal underwent three consecutive PET studies with [^18^F]FB-Gol1, [^18^F]FDG, [^18^F]FB-GR20 and 24 hours apart, each time accompanied by T1-, T1 with contrast enhancement and T2-weighted MR scans. During all scans, the animals were under anesthesia induced by an air mixture of 2% Isoflurane (Laboratorios Karizoo, Spain) and performed with VetEquip (USA) inhalation system. Initial sedation was performed in specialized introduction box, and then isoflurane-air mixture was supplied to the animals via mask directly into the scanners at the time of acquisition.

In the final stage of the experiments the animals were euthanized by overdosing of Zoletil (Virbac, France, 30mg/kg) and Xyla (Interchemie, Netherlands, 8mg/kg) injected i.p., and glioma tissue was collected for histological examination and determination of EGFR expression.

All experimental procedures were conducted in accordance with the European Communities Council Directive of 24 November 1986 (86/609/EEC) on the protection of animals used for scientific purposes. The study protocol was approved by the Ethics Committee of the Institute of Higher Nervous Activity and Neurophysiology of RAS (ethical approval № 4, 3rd of June 2025).

### 2.2 Magnetic resonance imaging

Before each PET examination, the rat underwent an MR- scanning of the brain region in order to localize the tumor focus for subsequent alignment and anatomic comparison with PET/CT data. MRI scans were made using a Mediso NanoScan 3T scanner (Hungary).

The T1-WI scan mode had the following parameters: The MRI sequence- spin-echo, projection type - axial, repetition time - 516 msec, echo time - 6.8 msec, field of view – 17 × 30 mm, spatial resolution - 0.21 × 0.21 mm, slice thickness- 1.0 mm, space between slices - 0.1 mm, the number of slices - 30. In the case of contrast enhancement, the gadolinium-based contrast agent (0.15 ml, Gadovist, Bayer) was injected into the tail vein of the rat before the start of the scan.

The T2-WI scan mode had the following parameters: The MRI sequence - spin-echo, projection type - axial, repetition time - 3000 msec, echo time – 111.2 msec, field of view – 17 × 30 mm, spatial resolution 0.21 × 0.21 mm, slice thickness – 1.0 mm, space between slices - 0.1 mm, number of slices - 30.

### 2.3 Radiopharmaceutical preparation

Preparation of [^18^F]FB-GR20 and [^18^F]FB-Gol1 conjugates was performed just before injection into the tail vein of a laboratory rat with the developed tumor. The detailed procedures of synthesis of the radiotracer 4-[^18^F]-fluorobenzylazide ([^18^F]FB) and preparation of solutions of labelled aptamers has been described previously (32).

The 5’-alkyne-modified aptamers used for conjugations were: Gol1, consisting of a 49 nucleotide sequence (GCC GGC ATT TTG ACG CCG CCC CGG CTG CTT ATG CTC CGG GGC ATA TGG C) and GR20, consisting of a 46 nucleotide sequence (ACG CAC CAT TTG TTG TTT AAT ATG TTT TTT TTT TTT AAT TCC TCC CCT TGT GGT GTG T) synthesized by GENTERRA Company (Moscow, Russia). Conjugations were performed using the Cu-catalyzed azide-alkyne cycloaddition (CuAAC) technique with tris(benzyltriazolylmethyl)amine (TBTA) premix with acetone reprecipitation in the presence of lithium perchlorate. The amount of aptamer required for conjugation was measured with Nanodrop One C spectrophotometer. The radiochemical purity of conjugates was determined by high-performance liquid chromatography (HPLC) on an Agilent 1290 Infinity II chromatograph equipped with a G7117B diode matrix detector and a Gabi Star radioactivity detector on a Phenomenex Luna C18 (2) 100Å (5 μm, 4.6×250 mm) column with gradient elution. Conjugates of [^18^F]FB-Gol1 and [^18^F]FB-GR20 with radiochemical purity better than 95% and an activity of at least 80 MBq per animal were used for the studies.

### 2.4 Micro-PET-CT study

The studies were performed on a preclinical PET-SPECT-CT imaging system MiLabs Vector6 (MiLabs, The Netherlands) and consisted of two stages: micro-PET and micro-CT scanning. A HE-UHR-RM collimator was used for PET scanning.

The radiopharmaceutical was injected intravenously into the tail vein of the animal 5 min prior to PET scanning. The administered doses were 96 ± 22 MBq, 121 ± 54 MBq, and 134 ± 63 MBq for [^18^F]FDG, [^18^F]FB-GR20 and [^18^F]FB-Gol1 preparations, respectively. The study area was selected on a topogram to completely cover the rat brain. The duration of data acquisition was 40 min (60 min in some animals), and 1 time frame was 4 min. Three animals were also injected with the radioactive precursor for aptamer conjugates 4-[^18^F]-fluorobenzylazide alone.

Micro-CT of the brain area was performed after PET data collection. The pixel size was 0.2 mm and the acquisition time was 150 seconds.

Slices of PET and CT studies were reconstructed using MiLabs Rec 12.00 software. The energy window for reconstruction was 511 ± 10% keV. Data was decay corrected for the injection time.

Post-processing, including qualitative and quantitative image analysis, was performed in PMod 4.1 (PMOD Technologies LLC, Switzerland). All PET-, CT-, and MR- images for all time points were spatially co-registered. Time activity curves of radio-pharmaceutical uptake in the tumor and healthy tissue were constructed. The metabolic volume of the tumor was estimated by the zone of increased radiopharmaceutical uptake relative to healthy tissue. The tumor volume was also measured using contrast-enhanced MRI data based on the volume of contrast agent accumulation.

The maximum SUV of the indicated regions of interest and the standard deviation were calculated to quantify aptamer uptake.

### 2.5 Histology

Brain samples were extracted from the cranial cavity and placed in 4% paraformaldehyde solution (pH=7.4). After 4–7 days, samples were sequentially incubated in ethanol and xylene solutions and encapsulated in paraffin. Sections 4-7 μm thick were stained with haematoxylin and eosin. Microscopic analysis was performed on a Leica DM 2500 microscope.

For immunohistochemical study, the animal was anesthetized by inhalation of isoflurane until breathing ceased, after which it was perfused through the heart with physiological saline followed by 4% formaldehyde solution in buffered physiological saline (PBS). The extracted brain was placed in the same fixative solution for 24-h additional fixation at +4 °C followed by soaking in 30% sucrose solution at the same temperature. Then, brains were frozen in a cryostat and 10 micrometer thick sections were made, which were mounted on slides, dried and stored at -20 °C until staining procedure. For immunohistochemical staining, slices were surrounded with a hydrophobic barrier using Pap pen (Abcam), wetted with PBS for 5 min, and then treated with a solution of primary mouse antibodies against EGFR (Affinity Biosciences, PRC) diluted 1/100 in PBS with 2% normal donkey serum and 0.5% Triton X-100, for 24 h at +4 °C. Then after washing in PBS, the sections were treated with a solution of donkey antibodies against mouse immunoglobulin conjugated with Cy2 fluorescent dye (Jackson ImmunoResearch, USA) diluted in PBS 1/100–1 h at room temperature. Stained sections were washed in PBS, cleared with glycerol and covered with a coverslip, and then examined and photo-graphed using a Nexcope NIB900 fluorescence microscope with a BestScope BUC51B-700M camera (PRC).

## 3 Results

**Figure 1:**
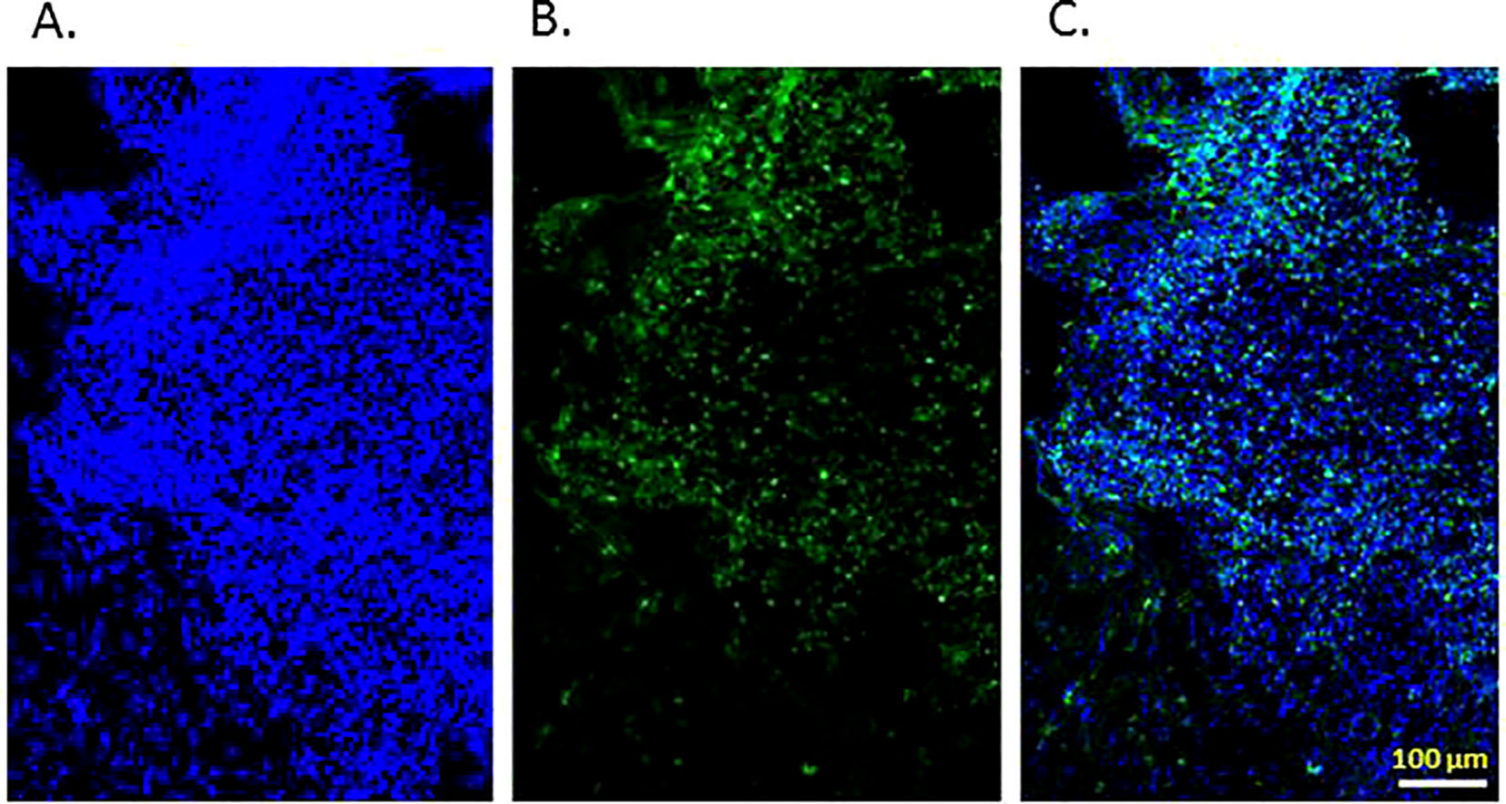
EGFR expression in rat 101.8 glioblastoma tissues (immunohistochemistry). **(a)** Staining of nuclei (bisbenzimide, Hoechst. Ge); **(b)** Staining with Cy2-labeled anti-EGFR antibodies (Jackson Immunoresearch, USA), **(c)** Combined image. The scale is 100 micrometers.

The rats with implanted tissue glioblastoma 101.8 were used to analyze radiopharmaceuticals conjugated with [^18^F]FB-Gol1 and [^18^F]FB-GR20 (32) aptamers and specific to EGFR distribution (14). Preliminary analysis on brain sections of model animals with tissue glioblastoma 101.8 by immunohistochemical investigation confirmed the expression of EGFR on tumor cells (**Figure 1**).

Of the 14 rats with implanted gliomas, 11 animals successfully completed all planned experiments. In one rat, the tumor did not grow to the required size, and two others did not tolerate anesthesia in one of the scans.

**Figure 2:**
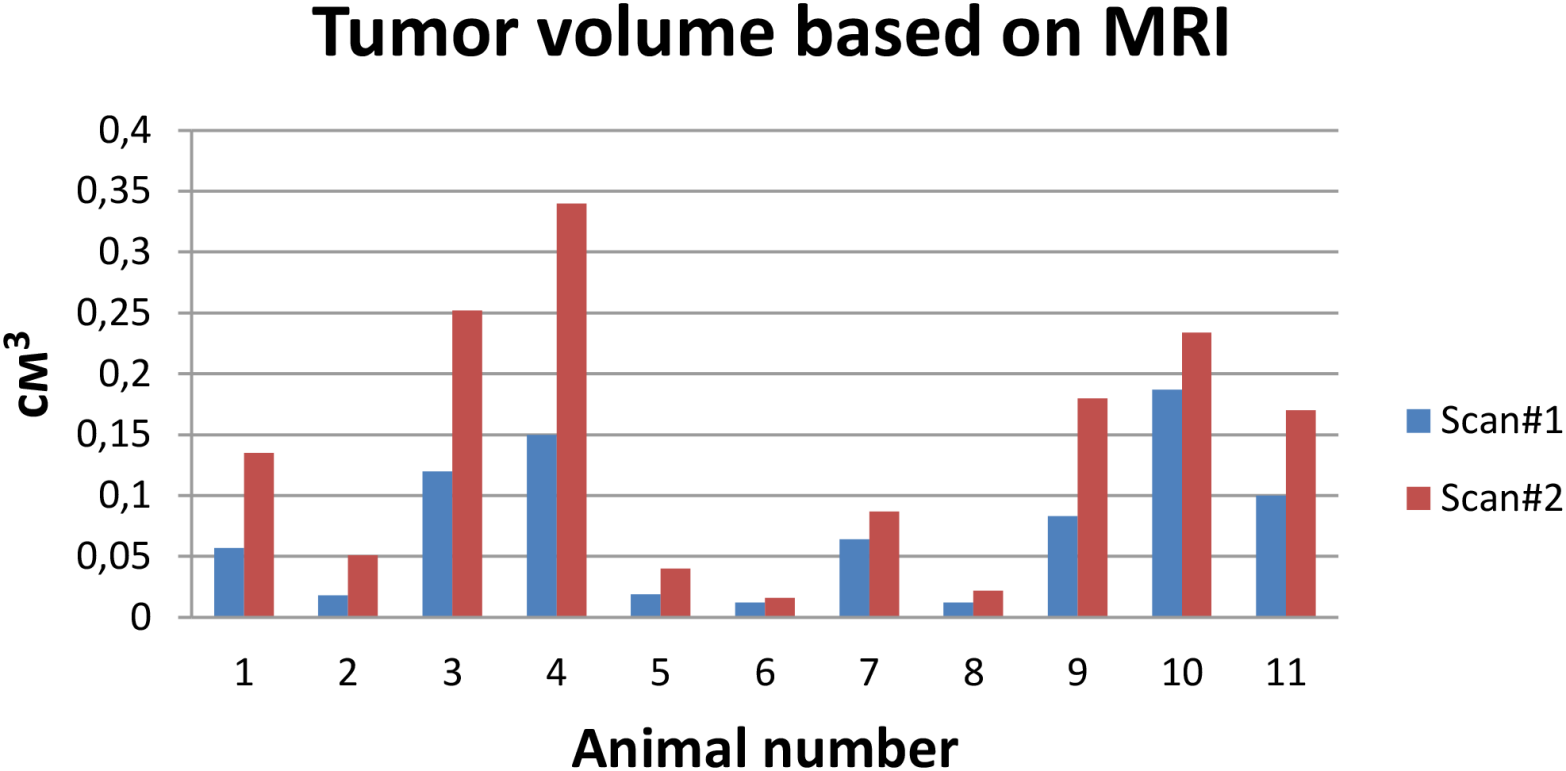
Comparison of the contrast enhancement volume on MRI on the time of [^18^F]-Gol1 and [^18^F]-GR20 acquisitions, 48 h apart.

To reduce the number of animals involved, both aptamers were supposed to be used on the same rats. Thus, a direct comparison of the radiotracers uptake efficiency was planned. However, despite the short interval between PET studies with aptamers (2 days), the tumor continued to develop actively. The tumor volume, measured by the contrasted volume on MRI, doubled between studies, amounting to 0.07 ± 0.06 cm^3^ in the first and 0.14 ± 0.11 cm^3^ in the second study (**Figure 2**). In this regard, the order of aptamer testing was alternated so that the distribution of tumor volumes became similar in each group and the aptamers were compared on groups of animals. Tumor development within 14–20 days after tumor tissue transplantation led to the formation of extensive necrosis and death of the animal.

**Figure 3:**
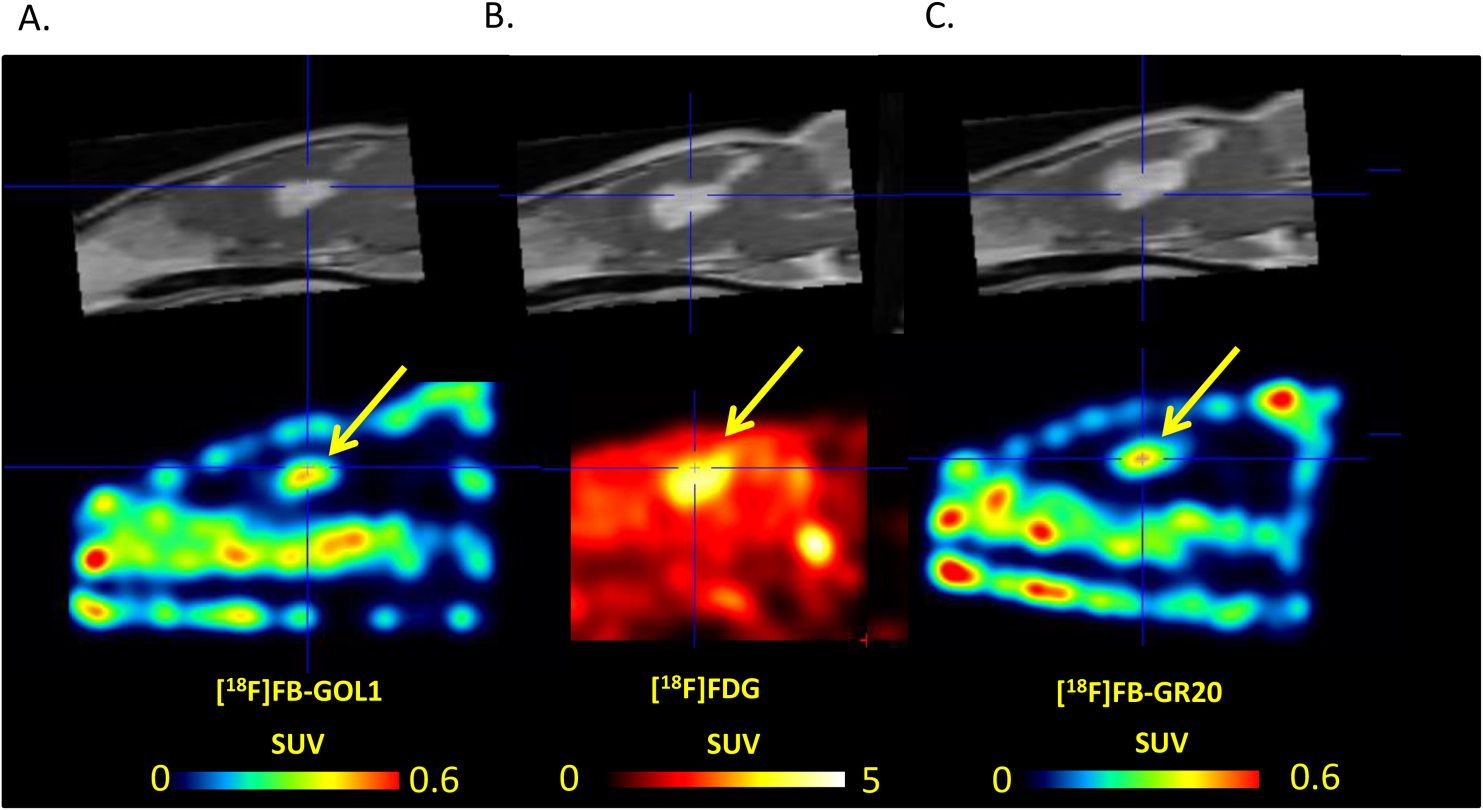
Comparison of tracers uptake **(a)** [^18^F]FB-Gol1; **(b)** [^18^F]FDG; **(c)** [^18^F]FB-GR20. The upper row is T1- MRI with contrast enhancement, the lower row is PET scans. The arrow points to the tumor.

The maximum standardized uptake values in the tumor were SUVmax_T=0.44 ± 0.22 and 0.43 ± 0.20 for [^18^F]FB-GR20 and [^18^F]FB-Gol1, respectively (no significant difference, p=0.91 for unpaired Student’s t-test). Both aptamers showed extremely low accumulation in healthy tissue (**Figure 3**), so that it was difficult to measure it reliably (SUVn was of the order of 0.02 for both drugs).

**Figure 4:**
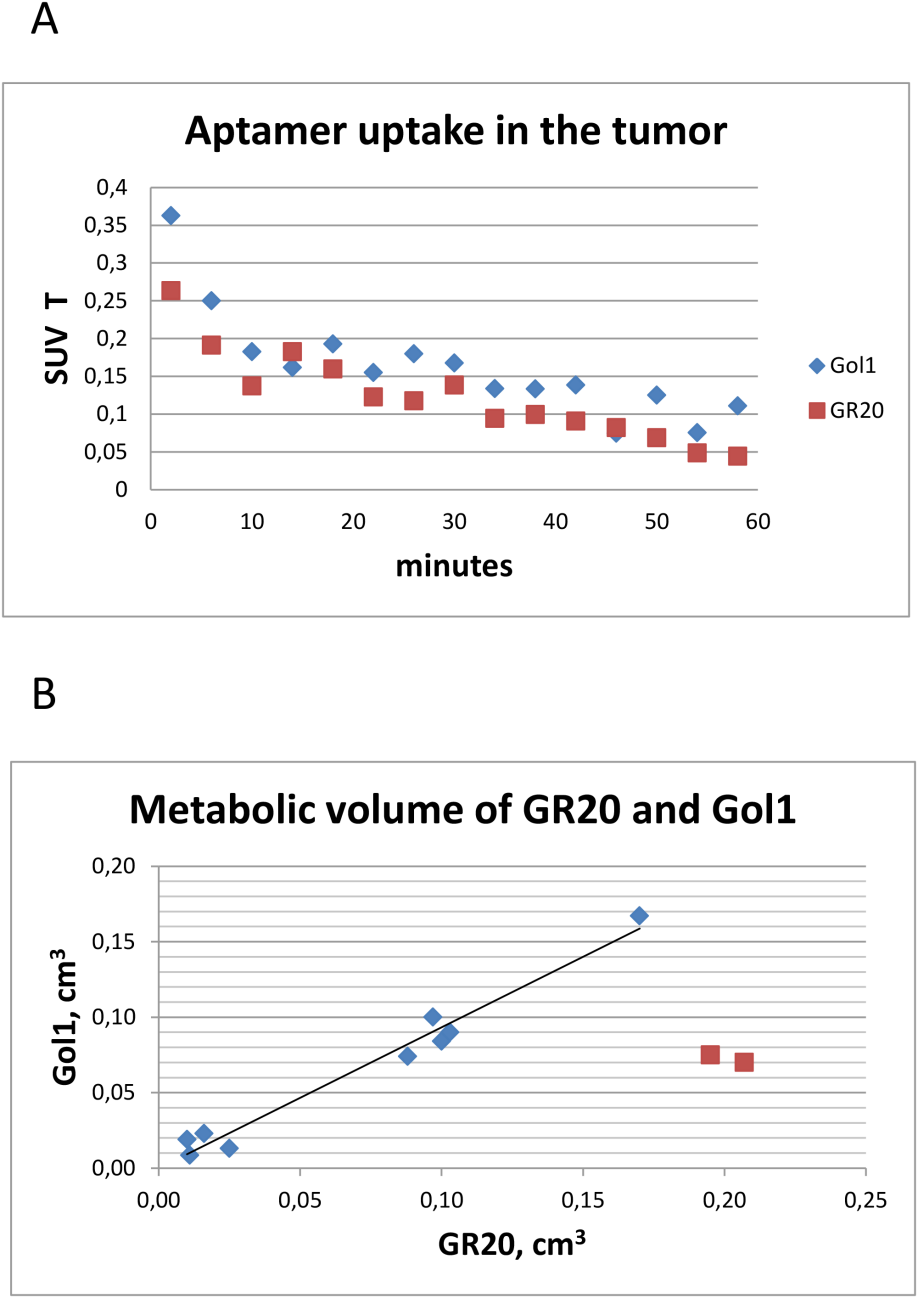
Characteristics of aptamer tracers uptake. **(a)** Averaged over all animals time activity curves for [^18^F]FB-Gol1 and [^18^F]FB-GR20; **(b)** Comparison of metabolic volumes of [^18^F]FB-Gol1 and [^18^F]FB-GR20. Two red dots indicate animals that developed extensive necrosis in the tumor between studies.

**Figure 5:**
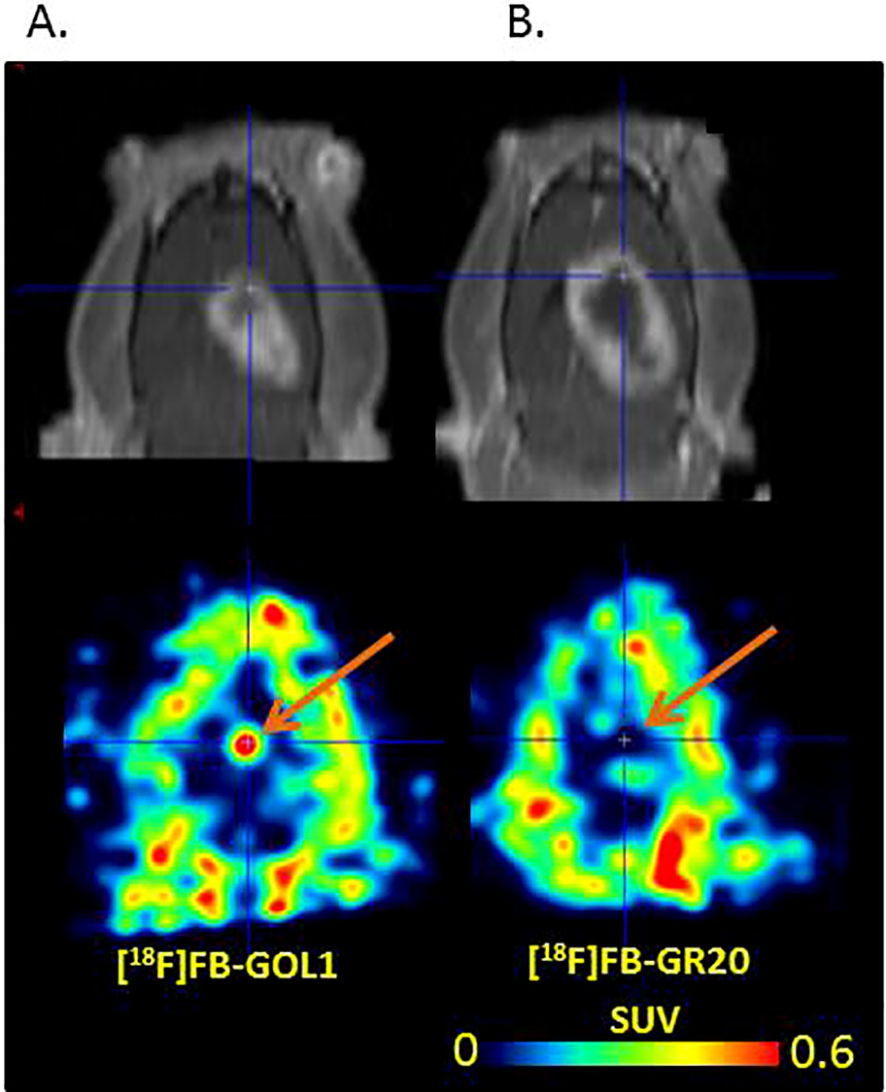
Formation of the intratumoral necrosis. Contrast enhanced T1-MRI combined with **(a)** [^18^F]FB-Gol1; **(b)** [^18^F]FB-GR20. The area of necrosis formation is pointed by the arrow on PET images.

The pharmacokinetics of the radiotracers appeared to be similar. Both aptamers [^18^F]FB-Gol1 and [^18^F]FB-GR20 were captured by the tumor within the first minutes after administration, within an hour the accumulation in the tumor dropped to 25% of the initial level (**Figure 4a**). Due to the negligible accumulation in the healthy part of the brain, it was possible to measure the metabolic volume of the tumor with near zero threshold value. The metabolic volume (**Figure 4b**) of GR20 was also equal to that of Gol1, 0.069 ± 0.056 cm^3^ versus 0.064 ± 0.053 cm^3^ when excluding the two animals that formed extensive necrosis during the time between studies (illustrated on **Figure 5**).

Studies with the reference radiopharmaceutical [^18^F]FDG showed the uptake in the structure of glioblastoma 101.8 (16), thereby demonstrating increased metabolic activity of the neoplasm. It was noted that in the presence of fluorodeoxyglucose accumulation in the tumor, both conjugates with aptamers also showed an increased degree of radio-pharmaceutical uptake in glioblastoma. At the same time, the metabolic volume of [^18^F]FDG accumulation was higher (0.088 ± 0.055 cm^3^) compared to both aptamer-containing radiopharmaceuticals. An interesting fact was the discrepancy between the volume of the contrast enhanced zone (**Figure 3**) according to MRI data (0.11 ± 0.10 cm^3^), which was almost in 2 folds more than the accumulation volume when using aptamers.

**Figure 6:**
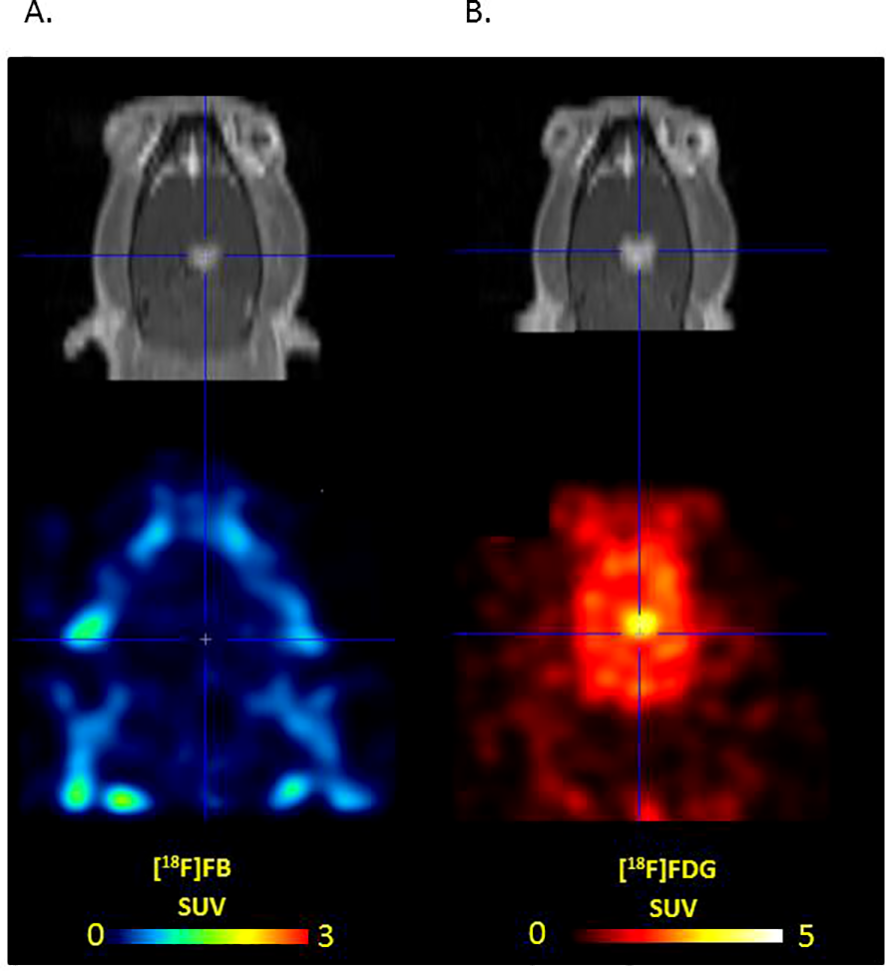
Unconjugated label uptake. T1 –MRI image with contrast enhancement and **(a)** [^18^F]-fluorobenzylazide and **(b)** [^18^F]FDG. While [^18^F]FB demonstrates no uptake, [^18^F]FDG accumulates in the lesion.

An additional part of our experiment was the study of the degree of accumulation of the radioactive label - 4-[^18^F]-fluorobenzylazide “free” from the aptamer within the zone of interest - in the brain and in the tumor. The study showed that the label, with its relatively uniform distribution in healthy brain tissue (with SUV of approximately 0.25), was not captured by tumor tissue in any of our three experimental animals that underwent these measurements (**Figure 6**).

## 4 Discussion

In this study, the objective was to investigate the uptake of EGFR-selective aptamers - Gol1 and GR20, labeled with a radioactive 4-[^18^F]- fluorobenzylazide, in laboratory animals with a grafted rat tissue 101.8 glioblastoma model.

The choice of EGFR as a target was due to the high expression of this receptor in a primary glioblastoma (observed in 60% of patients, EGFRvIII mutation in 50% of patients) (12), as well as the known negative effect of this biomarker on the prognosis of the disease.

In the work of Wu et al. in 2014 (18), DNA aptamers specific for the EGFRvIII receptor on U87-EGFRvIII cells were selected using the whole-cell systematic evolution of ligands by exponential enrichment (SELEX) method. Among them, the U2 and U8 aptamers demonstrated the highest affinity for EGFRvIII.

In 2018, Zhang et al. (13) confirmed that the U2 aptamer selectively bound to U87-EGFRvIII cells and penetrated them through endosomal recirculation, as determined by flow cytometry (FCM) and immunofluorescence analysis. In addition, 24-hour incubation of U87-EGFRvIII cells with U2 aptamer suppressed their proliferation, migration, and invasive activity, as well as stimulated apoptosis.

Later, a more stable Gol1 aptamer was created based on the U2 aptamer, which featured an optimized shortened structure to increase stability and efficiency (14). Competitive aptaimmunocytochemical staining of human glioblastoma cells overexpressing EGFRvIII (G-01/EGFRvIII) was carried out, which showed a higher competitiveness of the Gol1 aptamer binding to mutant EGFRvIII compared to commercial monoclonal antibodies to this type of receptor and the U2 aptamer (19). In addition, the study has shown that binding of the Gol1 aptamer to the EGFRvIII receptor leads to a change in the expression of key genes of the EGFR-mediated signaling pathways that regulate cell proliferation and survival. When performing the MTS test (a simplified and one-step MTT assay, using (3-(4,5-dimethylthiazol-2-yl)-5-(3-carboxymethoxyphenyl)-2-(4-sulfophenyl)-2H-tetrazolium)), Gol1 showed a significant antiproliferative effect on glioblastoma cell cultures, while the aptamer had no significant effect on normal cells, which confirms their specificity to tumor cells (14). Unlike Gol1, the GR20 aptamer, with comparable affinity, does not have an antiproliferative effect, which can be used under certain conditions (20).

Although rat glioma cell models [such as C6 (21)] are widely used for PET studies in the development of targeted drugs, to our knowledge, the presented work is the first to use the 101.8 tissue model, which engrafts and grows rapidly inside the brain of a laboratory rat. The development of this tumor is accompanied by high heterogeneity and is similar to the clinical and diagnostic picture observed in patients with glioblastoma (16, 22).

We are aware of only a few publications where an aptamer-based drug candidate has been tested using PET-CT. Click chemistry (23) using a fluorobenzylazide label, as in our study, was used to produce the labeled aptamer [^18^F]FB-ME07, which was tested in xenograft tumor models of A431, U87MG, and HCT-116 with different levels of EGFR expression. Micro-PET imaging confirmed that aptamer uptake was more pronounced in the model expressing more EGFR.

An aptamer specifically binding to the mutant EGFR ([^68^Ga]-NOTA-EGFRvIII) was tested in a xenograft mouse cell model of U87MG glioma expressing either EGFR or EGFRvIII that was injected subcutaneously and showed binding to EGFRvIII but no binding to conventional EGFR on micro-PET imaging (24).

However, the use of xenograft models does not provide an answer to how the radiopharmaceutical interacts with the tumor in the presence of the BBB, as well as in the case of its possible disruption. In our experiment, we demonstrated the absence of uptake of labeled aptamers in the intact part of the brain, with their effective accumulation in tumor tissue.

The use of substantial number of animals and estimates of SUV accumulation of both drugs allows for a better understanding of the diagnostic capabilities and features of the radiotracers, which cannot be performed on xenographic models. An important advantage of our work was the fact that the animals were studied at different stages of glioblastoma development, from the earliest period, where the aptamer can act as the earliest marker of the tumor process, and up to “giant” tumor sizes with the development of intratumoral necrosis (**Figure 5**).

The absence of accumulation of aptamer-containing radiopharmaceuticals in the intact brain matter of the animal allowed us to accurately measure the metabolic volume, determining the fact that any accumulation is due to the tumor process. At the same time, it should be noted that [^18^F]FB, administered in pure form, still accumulates in the healthy part of the brain, but without signs of uptake within the tumor structure defined with MRI with contrast enhancement.

In our opinion, the discrepancy between the MRI contrast enhanced volume and the metabolic volume recorded using aptamers in a number of our observations confirms that not the entire tumor is a target for [^18^F]FB-Gol1 and [^18^F]FB-GR20.

The limitations of the presented study are originated from the use of a universal PET-SPECT-CT tomograph, which has lower sensitivity and limitations in the temporal resolution of time-activity curves (TACs) compared to specialized micro-PET for animals. And despite the fact that during the experiments both aptamers showed similar kinetics and reversible binding, rapidly accumulating in the tumor, the pharmacokinetics of the drugs requires further detailed study.

A significant question is an estimation of whether these tracers could be translated for studies on humans. The SUVmax values of aptamers can be compared with the SUV values of [^18^F]-FET (SUVn=0.43 ± 0.16; tumor to brain ratio TBR about 2.5) and [^18^F]-FDG (SUVn=1.26 ± 0.20; TBR about 1.4) for the same rat 101.8 glioblastoma model (16), and they are also essentially lower than those observed in clinical settings (25). Therefore, expected SUV values of aptamers in patients could also be higher than values for rats. BBB penetration by aptamers is under intensive study, which has been recently reviewed (26).

Tracers that target specific tumor biomarkers, as in our study, already have applications in oncology. First of all we should mention the drugs that visualize hypoxic areas of the tumor ([^18^F]-FMISO (27), [^18^F]-EF5 (28)) and characterize resistance to radiotherapy.

It should be added that there are publications on the development of radiopharmaceuticals based on aptamers sensitive to the HER class of receptors, which significantly expands the capabilities of aptamer conjugates in general oncology. For example, an aptamer for the HER2 receptor was tested in a cell model of breast cancer using micro-PET (29). The use of a modern high-quality animal PET scanner (30) made it possible to evaluate the pharmacokinetics of the aptamer (in this publication, binding to ErbB2) based on a series of 3D images of the entire body of the experimental animal, as well as to perform dosimetry.

The aptamer [^68^Ga]-NOTA-SGC8 with a target for protein tyrosine kinase 7 (PTK-7) was clinically tested on volunteer patients (31) and showed no accumulation in the intestines, muscles, or brain, which is a favorable factor for the development of targeted diagnostics based on a new class of radiopharmaceuticals. The study also did not reveal high toxicity and did not demonstrate an increase in the dose load on humans.

## 5 Conclusions

This work demonstrates that radiopharmaceuticals [^18^F]FB-Gol1 and [^18^F]FB-GR20 bind to rat 101.8 glioblastoma and can serve as promising drug candidates for the selective diagnosis of EGFR expression in glial tumors.

## Data Availability

The original contributions presented in the study are included in the article/supplementary material. Further inquiries can be directed to the corresponding author.
